# Optimizing Microsatellite Marker Panels for Genetic Diversity and Population Genetic Studies: An Ant Colony Algorithm Approach with Polymorphic Information Content

**DOI:** 10.3390/biology12101280

**Published:** 2023-09-25

**Authors:** Ryan Rasoarahona, Pish Wattanadilokchatkun, Thitipong Panthum, Thanyapat Thong, Worapong Singchat, Syed Farhan Ahmad, Aingorn Chaiyes, Kyudong Han, Ekaphan Kraichak, Narongrit Muangmai, Akihiko Koga, Prateep Duengkae, Agostinho Antunes, Kornsorn Srikulnath

**Affiliations:** 1 Animal Genomics and Bioresource Research Unit, Faculty of Science, Kasetsart University, 50 Ngamwongwan, Bangkok 10900, Thailand; rasoarahonarivoniaina.h@ku.th (R.R.); pish.wa@ku.th (P.W.); thitipong.pa@ku.th (T.P.); thongthanyapat@gmail.com (T.T.); worapong.singc@ku.ac.th (W.S.); syedfarhan.a@ku.th (S.F.A.); jim97@dankook.ac.kr (K.H.); ekaphan.k@ku.th (E.K.); ffisnrm@ku.ac.th (N.M.); koga.aki.ku@gmail.com (A.K.); prateep.du@ku.ac.th (P.D.); 2 Sciences for Industry, Faculty of Science, Kasetsart University, 50 Ngamwongwan, Bangkok 10900, Thailand; 3 Special Research Unit for Wildlife Genomics, Department of Forest Biology, Faculty of Forestry, Kasetsart University, 50 Ngamwongwan, Bangkok 10900, Thailand; 4 School of Agriculture and Cooperatives, Sukhothai Thammathirat Open University, Pakkret Nonthaburi 11120, Thailand; chaiyes.stou@gmail.com; 5 Department of Microbiology, College of Science & Technology, Dankook University, Cheonan 31116, Republic of Korea; 6 Center for Bio-Medical Engineering Core Facility, Dankook University, Cheonan 31116, Republic of Korea; 7 Department of Botany, Faculty of Science, Kasetsart University, Bangkok 10900, Thailand; 8 Department of Fishery Biology, Faculty of Fisheries, Kasetsart University, Bangkok 10900, Thailand; 9 Interdisciplinary Centre of Marine and Environmental Research, University of Porto, Terminal de Cruzeiros do Porto de Leixões, Av. General Norton de Matos, s/n, 4450-208 Porto, Portugal; aantunes@ciimar.up.pt; 10 Department of Biology, Faculty of Sciences, University of Porto, Rua do Campo Alegre, s/n, 4169-007 Porto, Portugal; 11 Center for Advanced Studies in Tropical Natural Resources, National Research University, Bangkok 10900, Thailand

**Keywords:** ant colony optimization, microsatellite, marker selection, polymorphic information, population genetics

## Abstract

**Simple Summary:**

Microsatellite markers are widely used molecular markers for genetic studies, but choosing the right set involves a challenging trade-off between effectiveness and cost. The research aims to enhance the widely used ant colony optimization algorithm by integrating marker effectiveness indicators. By considering the genetic properties of the markers such as the polymorphic information content, the study seeks to determine the suitable way to select a reduced set of microsatellites. The approach addresses the accuracy–cost trade-off, aiding genetic assessments, breeding, and conservation efforts with cost-effective solutions. This research provides valuable insights into real-world genetic studies, including breeding programs and conservation initiatives.

**Abstract:**

Microsatellites are polymorphic and cost-effective. Optimizing reduced microsatellite panels using heuristic algorithms eases budget constraints in genetic diversity and population genetic assessments. Microsatellite marker efficiency is strongly associated with its polymorphism and is quantified as the polymorphic information content (*PIC*). Nevertheless, marker selection cannot rely solely on *PIC*. In this study, the ant colony optimization (ACO) algorithm, a widely recognized optimization method, was adopted to create an enhanced selection scheme for refining microsatellite marker panels, called the *PIC*–ACO selection scheme. The algorithm was fine-tuned and validated using extensive datasets of chicken (*Gallus gallus*) and Chinese gorals (*Naemorhedus griseus*) from our previous studies. In contrast to basic optimization algorithms that stochastically initialize potential outputs, our selection algorithm utilizes the *PIC* values of markers to prime the ACO process. This increases the global solution discovery speed while reducing the likelihood of becoming trapped in local solutions. This process facilitated the acquisition of a cost-efficient and optimized microsatellite marker panel for studying genetic diversity and population genetic datasets. The established microsatellite efficiency metrics such as *PIC*, allele richness, and heterozygosity were correlated with the actual effectiveness of the microsatellite marker panel. This approach could substantially reduce budgetary barriers to population genetic assessments, breeding, and conservation programs.

## 1. Introduction

Microsatellite repeats, also known as simple-sequence repeats, are abundant and highly polymorphic in numerous eukaryotic genomes. They represent a class of DNA markers with repeat sequences ranging usually from mononucleotides to hexanucleotide repeats. Perfect repetitions, interrupted repeats, or combinations with other repeat types are possible occurrences. Biparentally inherited nuclear DNA microsatellites enable diverse applications, including population characterization, origin determination, hybrid identification, and the assessment of inbreeding levels. Consequently, while genome-wide single-nucleotide polymorphisms (SNPs) are frequently employed in genetic studies related to populations, forensics, conservation, and evolution, it is worth noting that microsatellite genotyping may offer a greater degree of informativeness compared to biallelic SNP genotyping in several species. This heightened informativeness arises from the fact that microsatellites represent mutational hotspots, characterized by elevated levels of polymorphism and a larger allelic diversity within diverse populations [1,2,3,4]. The high polymorphism and Mendelian inheritance of microsatellites make them a good choice, with significant impacts on breeding programs and conservation efforts. The global utilization of microsatellite markers in local laboratories with low-cost investment is a practical alternative to SNP genotyping, which requires advanced equipment and technology. However, the number of suitable microsatellite loci, which ranges from 10 to 30, may vary depending on the study field and research group. To measure the level of genetic variation and inbreeding in indigenous chickens, 15–30 loci derived from FAO reference markers were used [5]. An interpretation bias arises when comparing data on diversity and identification owing to the utilization of a large, non-optimized marker panel. However, the use of such a panel does not guarantee accurate results and can lead to a significant waste of human and financial resources, ultimately resulting in biased outcomes. The precision and accuracy of every downstream process following genotyping are mainly dependent on the effectiveness of the microsatellite panel. Admittedly, while a larger number of loci logically provides more genetic information on a population, researchers must consider a compromise between result accuracy and cost-effectiveness by accounting for the margin of error and defined accuracy criteria.

The widely used ant colony optimization (ACO) algorithm is a heuristic, population-based, and bioinspired optimization method for solving combinatorial problems [6]. This concept was proposed by Colorni et al. [7]. By leveraging the inherent behaviors observed in ant colonies, the ACO algorithm aims to determine the optimal solution by considering a set of constraints or costs [8]. The selection of an optimal microsatellite panel is driven by the intricate relationship between the utilized loci and the inferred result, leading to the categorization of the problem as nonlinear programming [9]. Solving these problems becomes computationally aspirational, even when dealing with a reasonable number of microsatellite markers, owing to the existence of multiple discrete decision variables [10]. Similar methods have been proposed to address these problems, including the genetic algorithm [11], particle swarm optimization [12], traveling salesman [13], and ant colony algorithm [8], which correspond to the ACO algorithm. In each method, the resource consumption and underlying logic differ; however, they all display remarkable flexibility in resolving optimization problems across various research domains [14]. These algorithms identified suitable microsatellite marker sets without relying on prior genetic knowledge. However, owing to the stochastic nature of metaheuristic algorithms, a local solution, characterized by high accuracy, but not necessarily the optimal accuracy among all possibilities, may be discovered, which could be distant from the global solution [15].

In this study, we aimed to elucidate the critical accuracy/cost trade-off dilemma in population genetics research projects. Here, rather than using a raw heuristic optimization algorithm, the effect of incorporating polymorphic information on the algorithm’s performance was explored. We hypothesized that integrating a relevant effectiveness indicator of a marker set into the ACO algorithm can lead to valuable findings such as reduced computational time and improved accuracy in identifying the optimal solution. When selecting the optimal microsatellite panel, the accuracy indicator was used as the cost function to be maximized [16]. Several approaches have considered polymorphic information content (*PIC*) [17], matching probability [18], and gene variability [19] as accuracy indicators for microsatellite panels. Additionally, a genetic distance matrix was used to provide useful information for population structure estimation using a reduced set of microsatellites [20]. By conducting a comparative analysis, the impact of incorporating *PIC* as a decision variable in the algorithm was evaluated. Our approach can help address budgetary barriers to population genetic assessments, breeding, and conservation programs.

## 2. Materials and Methods

### 2.1. Refining an Intriguing Algorithm for Microsatellite Marker Selection

The microsatellite marker selection problem is characterized as a combinatorial search problem, where there is a search space *S* and a cost function *f* that must be minimized [10]. The search space *S* comprises all possible subsets of markers, totaling 2*^k^* potential solutions for *k* loci. Each subset was represented by a binary vector *I* = [*i*_1_, *i*_2_, …, *i*_n_], where i ∈ {0;1} indicated whether a specific microsatellite was included in the marker panel or not. The accuracy of a microsatellite marker panel on a given genotype dataset was quantified using the cost function *f*. The cost function *f* was determined by comparing the average genetic distance (AGD) between the full set of markers and the reduced set [10]. From a biological perspective, genetic distance is defined as the accumulated differences in alleles at each locus [20]. This was calculated based on the allelic frequencies observed from a given set of microsatellite markers using Equation (1). The genetic distance matrix was generated using the *dist* function implemented within the *adegenet* package in R version 4.2.2 [21].

(1) $$D\left(a,b\right)=-ln(\frac{\sum_{k=1}^{v}\sum_{j=1}^{m(k)}p_{aj}^{k}p_{bj}^{k}}{\sqrt{\sum_{k=1}^{v}\sum_{j=1}^{m(k)}{\left(p_{aj}^{k}\right)}^{2}}\sqrt{\sum_{k=1}^{v}\sum_{j=1}^{m(k)}{\left(p_{bj}^{k}\right)}^{2}}})$$

In this study, a marker selection algorithm was developed to effectively decrease the number of microsatellite markers used in population genetic studies. This was achieved by enhancing the ACO algorithm for marker selection [22] and utilizing *PIC* as an informative marker indicator [17,23]. The *PIC* for each microsatellite marker was calculated using the *PopGenUtils* package in R version 4.2.2 [21]. In the microsatellite selection scheme, loci were sorted based on their *PIC* and the highest-ranking microsatellite was integrated into the selected marker set.

### 2.2. Ant Colony Optimization Algorithm

The ACO algorithm was used to select an optimal set of microsatellite markers. The ACO algorithm, inspired by the natural behavior of ants, is a metaheuristic optimization technique [7]. To facilitate the application of the ACO algorithm, the search space was represented by a directed graph [24] with 2 × *N* nodes, where *N* denotes the total number of microsatellite loci [8]. The ant pheromones were randomly distributed along the pathways. During each iteration, the ants independently construct their solutions by probabilistically selecting pathways based on pheromone trails, which serve as indicators of the solution quality. Once all the ants have constructed their solutions, the pathways are sorted based on their quality, and the corresponding pheromone trails are updated. The ACO algorithm was then executed with the appropriate parameters to identify discriminant microsatellite loci (Table 1). Finally, the initial pheromone values were adjusted based on the *PIC* of each microsatellite marker. Microsatellites with high levels of polymorphisms were preferred to those with low levels. This approach aims to reduce the computational noise, minimize the number of required iterations, and avoid potential entrapment in local solutions [25]. The described panel optimization algorithms were implemented using a Python version 3.11 [26] script (File S1) and executed on a Linux Ubuntu server version 18.04 [27].

### 2.3. Microsatellite Marker Dataset

The microsatellite selection scheme was evaluated using two datasets obtained from genetic diversity studies: a chicken genotyping dataset and a Chinese goral genotype dataset. The chicken dataset, from the Siam Chicken Bioresource Consortium Project, encompassed 652 individuals, was analyzed using 28 marker loci and available from https://doi.org/10.5061/dryad.hhmgqnkm0 (accessed on 5 July 2023) [28,29,30,31]. The genotype information of 79 individuals across 11 markers in the Chinese goral dataset was downloaded from https://doi.org/10.5061/dryad.wstqjq2hm (accessed on 5 July 2023) [32,33]. The datasets used in this study were formatted using the GenAlEx tool version 6.51 [34] and were compatible with Microsoft Excel. The number of alleles per locus (*N*_a_), effective number of alleles (*N*_ea_), observed and expected heterozygosities (*H*_o_ and *H*_e_), and allele richness (*AR*) were evaluated for each microsatellite locus in both datasets. The *PIC* was computed using the “*PIC*” function available in the *polysat* package within R version 4.2.2 [35].

### 2.4. Comparative Evaluation of Marker Selection Schemes: ACO Algorithm, PIC, PIC + ACO, and Random Selection

A microsatellite marker selection model was fitted to minimize the loss of AGD accuracy. Four marker-sampling methods were used in this study. The first method employed in this study was the use of the ACO algorithm to select the most accurate panel without prior information regarding the polymorphisms of each locus. The second method involved sorting microsatellites based solely on their *PIC* and selecting the most informative loci. The third method involves ranking microsatellites based on their *PIC* and subsequently optimizing the set using *PIC* + ACO. A random selection scheme was used for the control group. Pairwise comparisons between selection schemes were conducted using the Tukey honest significance test, using the “pairwise_tukeyhsd” function from the *statsmodel* package [26]. The performance of each selection scheme was assessed through statistical pairwise comparisons using Tukey’s honest significance test. This analysis was conducted using the “pairwise_tukey_hsd” function from the *statsmodel* package in Python version 3.11 [26]. The *PIC* + ACO algorithm was used to progressively reduce the number of microsatellite markers to *N* = 2. The accuracy losses of the estimated values for *H*_o_, *H*_e_, and *AR* were evaluated. The AGD was reported, and graphical illustrations were generated using the “boxplot” function from the *matplotlib* package in Python version 3.11 [36]. Statistical regression analysis was conducted using the “OLS” function from the *statsmodel* package [37]. The estimation accuracy loss of *H*_o_ and *H*_e_ was determined by gradually reducing the number of microsatellite markers using the “plot” function from the *matplotlib* package in Python version 3.11 [36].

### 2.5. Estimation of Genetic Diversity Measurement on a Reduced Set of Microsatellite Markers

The microsatellite marker panel was assessed for each dataset by setting arbitrary error tolerances to 1%, 5%, and 10%. As a result, three reduced marker panels were created for chicken: GGA_1_ (1% error tolerance-reduced marker), GGA_5_ (5% error), and GGA_10_ (10% error), and three marker panels for Chinese goral: NGR_1_ (1% error), NGR_5_ (5% error), and NGR_10_ (10% error). The *N*_a_, *N*_ea_, *AR*, and *PIC* of the given population were evaluated in all microsatellite datasets, focusing on two statistical aspects: the mean difference between the measurements on the optimized and full sets, and the significance of the association of a higher measurement with the optimized set. The mean difference was used to explain the extent of deviation between the values reported for the full and reduced sets of microsatellites. The statistical *p*-value was calculated using an independent *t*-test and classified into four levels of significance: not significant (*p* > 0.05), slightly significant (0.01 < *p* < 0.05), moderately significant (0.001 < *p* < 0.01), and highly significant (*p* < 0.001). The statistical test was performed using the “ttest_ind” function from the *stats* package in Python version 3.11 [38]. The results were subsequently visualized using the “boxplot” function from the *matplotlib* package in Python version 3.11 [37]. The impact of reducing the number of microsatellites in a marker panel on population structure estimation was studied using three analytical methods: the Bayesian clustering algorithm [39], phylogenetic relationship analysis [40], and multidimensional scaling [41]. Population clustering analysis was conducted using Structure software version 2.3.4 [42]. The appropriate number of population clusters was determined by selecting the highest value of the Delta-*K* statistic, following the guidelines provided in the STRUCTURE software user manual [43]. The genetic distance between subpopulations was computed for the phylogenetic analysis using the “hclust” function from the *stats* package in R version 4.2.2 [35]. The dimensional scaling analysis was conducted using both principal component analysis (PCA) [44] with the “cmdscale” function from the *stats* package in R version 4.2.2 [35] and the discriminant analysis of principal components (DAPC). The resulting dimensional coordinates were visualized using the “dapc” function from the *adegenet* package in R version 4.2.2.

## 3. Results

### 3.1. Pairwise Comparison of Marker Selection Schemes on Two Genotype Datasets

The chicken and Chinese goral genotype datasets comprise *N*_a_ ranging from 5 to 82 alleles (average: 21), *N*_ea_ spanning from 1.14 to 26.22 (average: 6.40), *AR* ranging from 0.01 to 0.16 (average: 0.06), and *PIC* values ranging from 0.12 to 0.95 (average: 0.70) (Table S1). A comparison of the three selection methods indicated that the *PIC* + ACO selection scheme demonstrated superior accuracy on the chicken dataset for all marker quantities (*N*), except for *N* = 5 and *N* = 4, which showed statistical significance (*p* < 0.01). However, the ACO selection scheme was the most accurate for *N* = 5, whereas the *PIC* selection method showed the highest accuracy for *N* = 4. By contrast, for the Chinese goral dataset, the *PIC* + ACO scheme was the most accurate for marker sets consisting of nine, seven, and four loci. The highest accuracy was observed for marker sets comprising ten and eight microsatellites in the ACO scheme. However, for other values of *N*, higher accuracy was observed with randomly selected microsatellite markers than with the ACO, *PIC*, and *PIC* + ACO selection schemes (Tables S3 and S4; Figure S1).

### 3.2. Microsatellite Panel Selection Using Error Margins of 1%, 5%, and 10%

**Figure 1:**
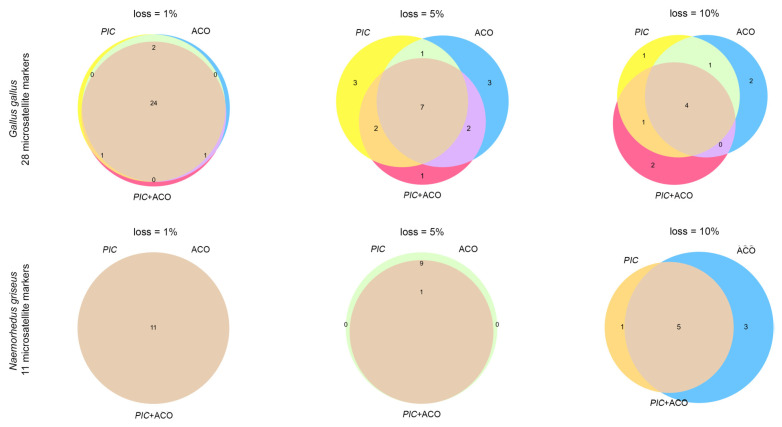
Microsatellite set reported by each of the 3 microsatellite markers selection scheme on the two datasets.

In the chicken dataset, with an error margin of 1%, the *PIC* + ACO selection method identified two microsatellites (LEI0094 and MCW0123) that could be excluded. Similarly, the ACO and *PIC* selection schemes each identified one microsatellite (MCW0206 and ADL0278, respectively) that could be excluded. With a permitted AGD estimation accuracy loss of 5%, the *PIC* + ACO selection scheme indicated the need for 12 marker loci. Based on the *PIC* selection policy, 13 markers were considered effective. The ACO selection algorithm required 13 markers, with 7 markers (MCW0034, MCW0183, LEI0192, MCW0123, LEI0234, MCW0069, and MCW0111) commonly selected by all three methods, including the ACO, *PIC*, and *PIC* + ACO selection schemes. Considering a threshold of 10% for AGD measurement, all three selection methods indicated the usability of 7 microsatellite markers, with 4 markers (LEI0234, MCW0104, LEI0192, and MCW0111) commonly selected by both methods. In the Chinese goral dataset, considering a 1% error allowance, all selection methods indicated that a full set of 11 markers was necessary. By selecting an error margin, the same set of markers consisting of 10 microsatellite markers, excluding SY259F, was reported by both the *PIC* and ACO selection schemes. In total, 9 microsatellite markers were identified as usable using the *PIC* + ACO selection method, excluding SY259F and SY128F. With an error margin of 10%, the ACO selection method determined that 8 microsatellite markers were adequate, excluding SY259F, SY76F, and SY449F. By contrast, the same set of 6 microsatellite markers (SY434F, SY14F, SY12BF, SY129F, SY449F, and SY128F) were identified using both the *PIC* and *PIC* + ACO selection schemes (Figure 1; Table 2).

### 3.3. Genetic Diversity Expressed by the Reduced Set of Microsatellites Using Error Margins of 1% (GGA_1_ and NGR_1_), 5% (GGA_5_ and NGR_5_), and 10% (GGA_10_ and NGR_10_)

Biased values of genetic diversity were observed between the full and reduced sets of microsatellites when employing the aforementioned markers, with varying levels of statistical significance and discrepancy. On the chicken dataset, the highest divergence in *N*_a_ was observed on the reduced set of microsatellites, which had an average of 26.88 alleles (1.02-fold higher than the full set of loci), 37.83 alleles (1.44-fold), and 48.14 alleles (1.83-fold) with the GGA_1_, GGA_5_, and GGA_10_ marker sets, respectively. Higher values of *N*_ea_ were observed on the GGA_5_ and GGA_10_ marker sets, with 10.97 (1.38-fold) and 12.6 (1.58-fold), respectively, whereas a negative discrepancy was observed in the GGA_1_ marker set, with an average *N*_ea_ of 7.49 (0.94-fold). Similarly, the GGA_1_ exhibited negative discrepancy in *N*_ea_, *AR*, *PIC*, *H*_o_, and *H*_e_: the measured *AR* was 0.04 (0.98-fold), *PIC* was 0.75 (0.95-fold), *H*_o_ was 0.59 (0.98-fold) and *H*_e_ was 0.82 (0.99-fold). Conversely, the GGA_5_ and GGA_10_ yielded relatively high values: their *AR* values were 0.06 (1.4-fold) and 0.08 (1.79-fold); their reported *PIC* 0.86 (1.07-fold) and 0.88 (1.12-fold); the determined *H*_o_ 0.66 (1.10-fold) and 0.68 (1.13-fold); and the *H*_e_ 0.88 (1.06-fold) and 0.90 (1.08-fold), respectively.

**Figure 2:**
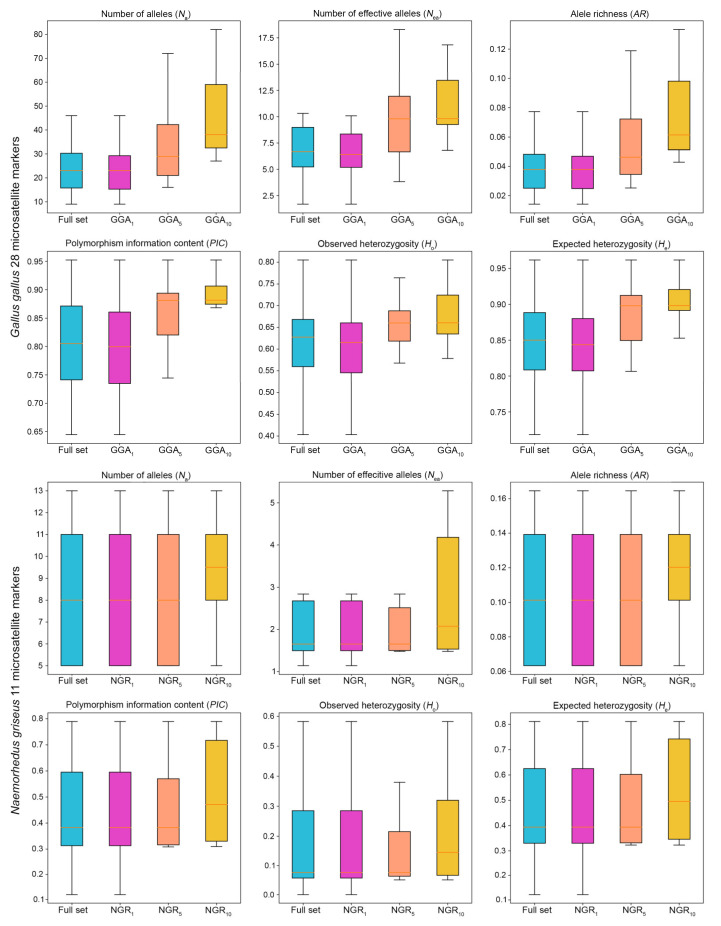
Measurement of the number of alleles (*N*_a_), the number of effective alleles (*N*_ea_), the allele richness (*AR*), the polymorphic information content (*PIC*), the observed (*H*_o_), and the expected heterozygosity (*H*_e_), comparatively calculated between the full set of microsatellites and the reduced set of microsatellite marker.

For the Chinese goral dataset, discrepancy analysis could only be performed for the NGR_5_ and NGR_10_ microsatellite sets because the NGR_1_ was not a reduced marker panel. The *N*_a_ allele exhibited an average of 8.66 alleles (1.01-fold) for NGR_5_ and 9.33 alleles (1.09-fold) for NGR_10_. The *N*_ea_ averaged a value of 2.27 (0.94-fold) for NGR_5_ and 2.86 (1.19-fold) for NGR_10_. The *AR* averaged a value of 0.11 (1.01-fold) for NGR_5_ and 0.11 (1.09-fold) for NGR_10_. The *PIC* yielded an average value of 0.46 (1.01-fold) for NGR_5_ and 0.52 (1.14-fold) for NGR_10_. *H*_o_ averaged a value of 0.16 (0.87-fold) for NGR_5_ and 0.22 (1.21-fold) for NGR_10_. The *H*_e_ yielded an average value of 0.48 (1.01-fold) for NGR_5_ and 0.54 (1.13-fold) for NGR_10_ (Figure 2; Table S2).

Previously described values were used to demonstrate the correlation between microsatellite panel quality and population genetic measurements at different levels of significance. In the GGA_5_ marker panel, moderately significant associations (*p* < 0.01) were observed for *N*_a_, *N*_ea_, and *AR*, and low statistical significance (0.01 < *p* < 0.05) was determined for *PIC*, *H*_o_, and *H*_e_. For GGA_10_, *N*_a_ and *AR* were determined to have high statistical significance (*p* < 0.001), *N*_ea_ exhibited moderate statistical significance (0.001 < *p* < 0.01), *PIC* and *H*_e_ had low statistical significance (0.01 < *p* < 0.05), and *H*_o_ had no statistical significance. However, for the chicken GGA_1_ and Chinese goral datasets (NGR_1_, NGR_5_, and NGR_10_), insufficient data used for the statistical tests hindered the achievement of statistically significant findings (Table 3).

### 3.4. Comparison of Population Structure Inference between the Full Set and Reduced Sets of Microsatellites

**Figure 3:**
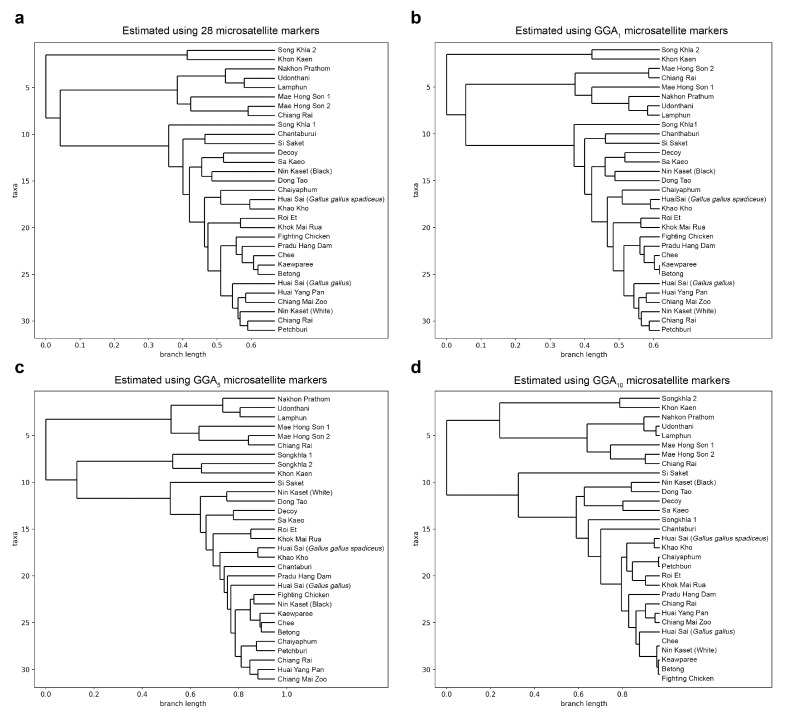
Phylogenetic relationship of the chicken population estimated using the full set of 28 microsatellites (**a**), the GGA_1_ (**b**), the GGA_5_ (**c**), and the GGA_10_ (**d**) reduced marker panels.

The presence of two population clusters (*K* = 2) was revealed in the downstream analysis of the chicken population genotype dataset using STRUCTURE software. Regardless of the number of microsatellite markers used for the population genetics assessment, the same value of *K* = 2 was consistently observed (Table S4; Figure S2). Visualization of population genetics and microsatellite marker panel accuracy can be achieved using STRUCTURE, phylogenetic trees, PCA, and DAPC plots (Figure 3, Figures S3 and S4). All 31 chicken subpopulations were classified into *K* = 2 clusters with statistical significance for the posterior probability (*p* < 0.01) for the four studied marker panels (GGA_1_, GGA_5_, GGA_10_, and the full set of 28 chicken microsatellites). For *K* = 7, 28 of the 31 subpopulations were successfully clustered into 7 groups using the full set of 28 microsatellites with statistical significance (*p* < 0.01). With GGA_1_, the number of clustered subpopulations remained at 28, whereas GGA_5_ clustered 29 subpopulations and GGA_10_ 26 subpopulations. For *K* = 9, 30 out of 31 subpopulations were assigned to 9 clusters using the full set of 28 markers, whereas both the GGA_1_, GGA_5_, and GGA_10_ marker panels reported 29 clustered subpopulations (Figure 3; Table S5). However, with the use of a reduced set of microsatellite markers, different values were reported, and no inferred clusters were revealed in the membership probability structure, PCA, and DAPC analysis. Because there was only one genetic subpopulation in the Chinese goral dataset, no statistical comparison of subpopulation clustering could be inferred.

## 4. Discussion

Genetic researchers face the challenge of an increasing number of usable microsatellite panels, prompting the need for smart and efficient selection of markers in the fields of genetic diversity, population genetics, and breeding programs. A trade-off between cost and result quality must be made, considering research expenses and time as limiting factors. In previous studies, various marker selection algorithms have been investigated, including the k-optimal [45], decision-tree induction algorithm [46], traveling salesman [13], ant colony algorithm [8], and genetic algorithm [11]. Considering panel selection as an optimization problem, any of the previously studied algorithms can be used as they offer a cost function to minimize or maximize [16].

### 4.1. Challenges in Microsatellite Marker Panel Selection

The informativeness of microsatellite markers is directly related to their degree of polymorphism [17]. The polymorphism exhibited by each marker (locus) should be considered when constructing a microsatellite panel [47]. A reduced panel of 9–12 markers was considered suitable. However, in genetic diversity and population analyses of species such as chickens, cattle, and dogs, the use of 18–30 markers is common. These species, which are known for their numerous varieties and breeds, have been studied and improved through breeding programs using microsatellite standard sets. However, considerable variations have been observed in the effectiveness and accuracy of each available microsatellite marker panel. The quality of the results is largely dependent on the choice of the marker set, as not all microsatellite panels are equivalent [48,49]. Usable and convenient microsatellite markers can be identified by combing through past studies; however, a universal optimized marker panel does not exist because of the varying genetic marker specifications across different research domains [50,51]. Another method uses the *PIC*, allele variation (*N*_a_/*N*_e_), *AR* and *H*_e_ as informativeness indicators of a particular locus [49,52]. The use of a well-selected panel could also compensate for certain genotyping errors and estimate population genetic measurements within an acceptable accuracy loss [10,53].

The *PIC* has always been regarded as an accurate quality indicator of microsatellite markers; however, the developed selection scheme does not prioritize the highest *PIC* microsatellites [17,23]. With the chicken dataset, of the reported 7-microsatellite set, LEI0094 and MCW0123, despite having high *PIC* values—0.93 and 0.88—respectively, were excluded. Instead, our marker selection scheme (*PIC* + ACO) included MCW0183 and MCW0016, which have *PIC* values, of 0.83 and 0.87, respectively. Similarly, among the 14 microsatellite marker sets, MCW0016, MCW0295, MCW0330, and ADL0268 (with *PIC* values of 0.87, 0.84, 0.85, and 0.85, respectively) were excluded, whereas LEI0166, MCW0165, and MCW0206 (with *PIC* values of 0.74, 0.69, and 0.81, respectively) were selected. This suggests that the accuracy of individual identification is not always guaranteed by the highest *PIC* markers, as microsatellite markers can provide redundant information due to non-random associations between distant loci [54]. However, regardless of the chosen accuracy loss threshold, all markers with low *PIC* values are generally excluded by the *PIC* + ACO selection scheme, with an allowed accuracy loss of 10%, all markers with *PIC* lower than 0.83 are excluded, and a loss tolerance of 5% excludes all markers with *PIC* below 0.69. This suggests that *PIC* provides valuable insights into the efficiency of molecular markers for genetic studies, as stipulated by Serrote et al. [17]. Publicly available microsatellite panels for genetic studies and chicken breeding programs are generally highly polymorphic [5,28,29,30,31]. Similarly, in the second dataset, the same set of markers was reported using the *PIC* and *PIC* + ACO selection schemes for margin tolerances of 1% and 10%, respectively. However, with a 5% margin tolerance, *PIC* + ACO excluded SY128F, which was among the top two highest *PIC* microsatellites in the dataset. In addition, the highest *PIC* markers were always selected by the *PIC* + ACO method for 1% and 10% error tolerances. Referring to the chicken dataset used in this study, an average genetic distance accuracy loss ranging from 5% (GGA_5_) to 10% (GGA_10_) was observed. The chicken genotype dataset revealed that the 7 most informative microsatellites were MCW0111, LEI0234, MCW0034, MCW0016, LEI0192, MCW0183, and MCW0104 markers. These markers exhibited higher effectiveness (*PIC* > 0.83, *N*_a_ > 28, *N*_ea_ > 6.79, *H*_o_ > 0.58, and *H*_e_ > 0.85), as suggested by previous studies on chicken population genetics [30,55]. Moreover, the clustering of the putative chicken population was accurately displayed by visual representations of PCA and DAPC using the 7 selected markers mentioned above. Microsatellite marker set reduction could be further pursued by increasing the accuracy loss margin by up to 15%, as reported by Xiong et al. [54] for other types of molecular markers. The relevance of the proposed microsatellite panel size was further supported by experiments on the Chinese goral dataset, which did not yield any marker combination with fewer than 9 markers (NGR_5_).

Microsatellite panels with high levels of genetic diversity are widely available for numerous species, therefore expanding the applicability and scope of this study [28,56]. The algorithm studied was well-suited for refining a large set of microsatellites (more than 20 microsatellite sets) with sufficient alleles to allow for some accuracy loss in the genetic measurement estimations. Using this algorithm, significant budgetary savings can be achieved by excluding a substantial number of microsatellite markers. Moreover, valuable insights into the efficiency of microsatellites and their individual contributions to the effectiveness of marker panels can be obtained [47]. However, the heterozygosity of individuals is not considered by the AGD function used to assess genetic diversity among populations [20], causing the algorithm to disregard valuable information on gene diversity and inbreeding within populations. Moreover, failures during microsatellite marker amplification and genotyping processes have been omitted in almost all studies [57], potentially leading to the exclusion of some usable microsatellite markers for population genetic investigation [58].

### 4.2. Using the PIC as a Discriminative Power Indicator of the Marker

The ant colony optimization (ACO) algorithm, which was proposed in the early 90s as an approach to resolving optimization problems, has garnered interest because of its simplicity and versatility [7]. It exists in numerous variants, including the ant system (AS), ant-Q, max-min ant system, rank-based ant system, BWAS, and hypercube AS [59,60,61,62]. The ACO algorithm, which belongs to the group of metaheuristic approaches [14], shares commonalities with trending optimization algorithms, such as the genetic algorithm (GA), particle swarm (PSO), or seagull optimization algorithm (SOA). It determines the optimal solution by spreading pheromones on pathways based on the solution quality [8]. Properly balancing exploration and exploitation in the algorithm parameters is crucial to avoid infinite loops or becoming stuck in local solutions [7]. Similar to the trial and reward concept used in reinforcement learning, every possibility of the microsatellite panel was assessed using the optimization pipeline used in the ant colony optimization algorithm, and a quality score was assigned to each based on certain criteria [63]. The original version of the ant colony optimization algorithm formulated by Colorni et al. [7] used a stochastically generated initial solution that was gradually improved. However, the discriminative power of markers is closely related to various variables, including *N*_a_, *N*_ea_, *AR*, and *PIC* [17,20]. This led to the investigation of a method that includes this information as an initial variable to be progressively improved by the heuristic algorithm. For the chicken dataset, a comparative study of the four selection schemes revealed that the accuracy of the improved algorithm (*PIC* + ACO scheme) was higher than that of the original algorithm (ACO). With the optimized chicken microsatellite and 5% accuracy loss, 3 highly polymorphic markers (MCW0104, LEI0094, and LEI0166) were omitted by ACO but included in the GGA_5_ panel.

### 4.3. Implications for Conservation Effort and Breeding Program

The chicken and Chinese goral datasets used in this study were sufficiently large to facilitate the use of the marker optimization algorithm [28,29,30,31,32,33]. The availability of a large genotype dataset allows for a more optimized exploration of the marker efficiency mechanism. In addition to the widely developed non-invasive sampling methods [64], the assessment and elucidation of genetic diversity can be significantly enhanced by the development of molecular markers. Population dynamics and migration in several animals have been studied using non-invasive fecal sampling [65]. However, the quality of the DNA stock after extraction is very low, and not all common sets of microsatellite genotyping are applicable. The competency of the output results in the full set can be effectively predicted by optimizing the microsatellite marker panel. Conservation and breeding initiatives can be greatly enhanced by the in silico development of microsatellite markers, enabling a more optimized fit for the proposed microsatellite panel reduction scheme presented in this study [66]. Budgetary barriers to numerous conservation and breeding initiatives would be considerably alleviated by this approach, offering an opportunity for population monitoring within an acceptable accuracy loss in conservation and breeding programs. Interestingly, the number of markers that can be amplified in a single reaction significantly influences both cost and efficiency. This relationship offers opportunities for cost reduction. Although marker multiplexing effectively manages this trade-off, PCR efficiency is not closely tied to polymorphism. In our current study, we prioritize polymorphism, leaving the amplification efficiency of markers as a potential focus for future research.

## 5. Conclusions

This study explored the use of a modified ACO algorithm, *PIC* + ACO selection scheme, to determine the most effective microsatellite panel for genetic diversity research with different accuracy loss tolerances. Experiments on both datasets revealed that microsatellite markers allow for the exclusion of many markers while maintaining acceptable precision in population genetics assessment. The optimized reduced set of markers exhibited efficiency related to various metrics. However, the *PIC* + ACO selection scheme shows that markers rely on hidden variables beyond simple metrics. The study results show that reducing laboratory costs could promote conservation initiatives and population genetic investigations in biodiversity conservation and breeding programs for genetic improvement.

## Figures and Tables

**Table 1:** Parameter used for the ant colony optimization algorithm [7,8].

Parameter	Description	Value
*ant_n*	Ant population size	50
*E*	Number of epochs (iterations)	120
*α* ^1^	Weight factor of the pheromone trail in the decision-making process	0.7
*decay* ^2^	Evaporation rate of the pheromone trail	0.9

^1^ A higher value of α increases the significance of the pheromone trail, making the ants more likely to choose edges with stronger pheromone concentrations. ^2^ A small value of *decay* allows the avoidance of becoming stuck on local minima and the encouragement of ants to explore new pathways.

**Table 2:** Microsatellite marker panel selected by the 3-selection scheme using different accuracy loss margins.

Dataset	Average Genetic Distance Estimation Accuracy Loss		Selection Scheme	
		*PIC* + ACO ^1^	ACO ^2^	*PIC* ^3^
*Gallus gallus* 28 markers	10%	MCW0034, MCW0104, LEI0234, MCW0016, MCW0111, MCW0183, LEI0192	MCW0104, LEI0234, LEI0166, MCW0123, MCW0111, ADL0268, LEI0192	MCW0034, MCW0104, LEI0234, MCW0123, MCW0111, LEI0094, LEI0192
	5%	MCW0034, MCW0104, MCW0165, LEI0234, MCW0123, MCW0206, MCW0111, LEI0094, MCW0183, MCW0069, LEI0166, LEI0192	MCW0034, MCW0078, MCW0098, MCW0165, LEI0234, MCW0216, MCW0123, MCW0206, MCW0111, MCW0183, MCW0069, ADL0268, LEI0192	MCW0034, MCW0104, MCW0330, LEI0234, MCW0123, MCW0016, MCW0111, LEI0094, MCW0183, MCW0069, MCW0295, ADL0268, LEI0192
	1%	MCW0034, MCW0098, MCW0081, MCW0330, MCW0165, LEI0234, MCW0222, MCW0206, MCW0104, MCW0078, ADL0112, MCW0216, MCW0111, MCW0183, MCW0069, ADL0268, LEI0192, MCW0037, MCW0248, MCW0014, MCW0103, MCW0067, MCW0016, MCW0295, LEI0166, ADL0278	MCW0034, MCW0098, MCW0081, MCW0330, MCW0165, LEI0234, MCW0222, MCW0104, MCW0078, ADL0112, MCW0216, MCW0111, MCW0183, MCW0069, ADL0268, LEI0192, MCW0037, MCW0248, MCW0014, LEI0094, MCW0103, MCW0067, MCW0123, MCW0016, MCW0295, LEI0166, ADL0278	MCW0034, MCW0098, MCW0081, MCW0330, MCW0165, LEI0234, MCW0222, MCW0206, MCW0104, MCW0078, ADL0112, MCW0216, MCW0111, MCW0183, MCW0069, ADL0268, LEI0192, MCW0037, MCW0248, MCW0014, LEI0094, MCW0103, MCW0067, MCW0123, MCW0016, MCW0295, LEI0166
*Naemorhedus griseus* 11 markers	10%	SY434F, SY14F, SY12BF, SY129F, SY449F, SY128F	SY434F, SY14F, SY12BF, SY129F, SY449F, SY128F	SY434F, SY14F, SY12BF, SY93F, SY129F, SY128F, SY84BF, SY84F
	5%	SY434F, SY14F, SY12BF, SY93F, SY129F, SY76F, SY449F, SY84BF, SY84F	SY434F, SY14F, SY12BF, SY93F, SY129F, SY76F, SY449F, SY128F, SY84BF, SY84F	SY434F, SY14F, SY12BF, SY93F, SY129F, SY76F, SY449F, SY128F, SY84BF, SY84F
	1%	SY434F, SY14F, SY259F, SY12BF, SY93F, SY129F, SY76F, SY449F, SY128F, SY84BF, SY84F	SY434F, SY14F, SY259F, SY12BF, SY93F, SY129F, SY76F, SY449F, SY128F, SY84BF, SY84F	SY434F, SY14F, SY259F, SY12BF, SY93F, SY129F, SY76F, SY449F, SY128F, SY84BF, SY84F

^1^ *PIC* + ACO, selection scheme involving ranking the markers by their polymorphic information content and subsequently optimizing the set using the *PIC* + ACO algorithm. ^2^ ACO, selection scheme using only the ant colony optimization algorithm without any prior information on the *PIC* of the markers. ^3^ *PIC*, selection scheme sorting microsatellites on their *PIC* and selecting the most informative loci.

**Table 3:** Statistical significance of the association of the number of alleles (*N*_a_), the number of effective alleles (*N*_ea_), the allele richness (*AR*), the polymorphic information content (*PIC*), the observed (*H*_o_), and the expected heterozygosity (*H*_e_) with the reduced microsatellite marker panel.

Dataset	Reduced Panel	Measurement	Mean-Diff	*t*-Stat	*p*-Val	Significance
*Gallus gallus* 28 markers	GGA_1_ (26 markers)	*N*_a_	5.115	−0.394	0.697	ns
		*N*_ea_	6.813	−1.909	0.067	ns
		*AR*	0.008	−0.397	0.695	ns
		*PIC*	0.122	−1.341	0.192	ns
		*H*_o_	0.101	1.975	0.108	ns
		*H*_e_	0.099	2.354	0.193	ns
	GGA_5_ (12 markers)	*N*_a_	18.521	3.240	0.003	**
		*N*_ea_	5.246	3.093	0.005	**
		*AR*	0.030	3.146	0.004	**
		*PIC*	0.110	2.515	0.018	*
		*H*_o_	0.105	2.422	0.023	*
		*H*_e_	0.086	2.347	0.027	*
	GGA_10_ (7 markers)	*N*_a_	27.857	5.081	0.000	***
		*N*_ea_	6.175	3.222	0.003	**
		*AR*	0.045	4.866	0.000	***
		*PIC*	0.129	2.586	0.016	*
		*H*_o_	0.101	1.975	0.059	ns
		*H*_e_	0.099	2.354	0.026	*
*Naemorhedus griseus* 11 markers	NGR_1_ (11 markers)	*N*_a_	–	–	–	–
		*N*_ea_	–	–	–	–
		*AR*	–	–	–	–
		*PIC*	–	–	–	–
		*H*_o_	–	–	–	–
		*H*_e_	–	–	–	–
	NGR_5_ (9 markers)	*N*_a_	0.667	0.251	0.808	ns
		*N*_ea_	0.668	−0.595	0.567	ns
		*AR*	0.008	0.228	0.825	ns
		*PIC*	0.015	0.087	0.933	ns
		*H*_o_	0.130	−0.899	0.392	ns
		*H*_e_	0.026	0.147	0.886	ns
	NGR_10_ (6 markers)	*N*_a_	1.733	0.874	0.405	ns
		*N*_ea_	1.022	1.249	0.243	ns
		*AR*	0.023	0.892	0.396	ns
		*PIC*	0.142	1.135	0.286	ns
		*H*_o_	0.087	0.771	0.460	ns
		*H*_e_	0.140	1.081	0.308	ns

ns: No significant association (*p* > 0.05). *: Weak significance association (0.05 < *p* < 0.01). **: Medium significance association (0.01 < *p* < 0.001). ***: High significance association (*p* < 0.01).

## Data Availability

The genotype data used in this project are publicly available on https://doi.org/doi:10.5061/dryad.hhmgqnkm0 (*Gallus gallus* genotype dataset, accessed on 5 July 2023) and https://doi.org/10.5061/dryad.wstqjq2hm (*Naemorhedus griseus* dataset, accessed on 5 July 2023).

## Supplementary Materials

The following supporting information can be downloaded at: https://www.mdpi.com/article/10.3390/biology12101280/s1. File S1: Python implementation of ant colony optimization algorithm for selection of an optimized microsatellite marker panel; Figure S1: Accuracy comparison of four microsatellite marker schemes including the ant colony optimization (ACO), the selection by polymorphic information content (*PIC*), and hybrid method consisting by optimizing the most informative set via ACO (PIC + ACO), and a random selection used as a control group; Figure S2: Population structure estimation of the chicken using the full set of 28 microsatellite markers (a), the GGA1 (b), the GGA5 (c) and the GGA10 (d) reduced set of microsatellite; and the Chinese goral using the full set of 11 microsatellite markers (e), the NGR1 (f), NGR5 (g) and NGR10 (h) optimized marker panel; Figure S3: Principal component analysis (PCA) plotting of the population structure estimation of the chicken using the full set of 28 microsatellite markers (a), the GGA1 (b), the GGA5 (c) and the GGA10 (d) reduced set of microsatellites; and the Chinese goral using the full set of 11 microsatellite markers (e), the NGR1 (f), NGR5 (g) and NGR10 (h) optimized marker panel; Figure S4: Discriminant analysis of principal component (DAPC) plotting of the chicken population using full set of 28 microsatellite markers (a), the GGA1 (b), the GGA5 (c), and the GGA10 (d) reduced set of microsatellites; Table S1: Summary of microsatellite markers used in this study; Table S2: Summary of microsatellite markers selected by the PIC + ACO selection scheme according to various margin errors. Data include number of alleles (Na), effective number of alleles (Nea), allele richness (AR), polymorphic information content (PIC), and observed (Ho) and expected heterozygosity (He); Table S3: Statistical comparison between the most accurate selection method and the random microsatellite selection scheme; Table S4: Number of population cluster estimated by the Structure software (Evanno et al., 2005 [43]); Table S5: Clustering of each subpopulations using the Bayesian clustering of the Structure software (Evanno et al., 2005 [43]).
