# Supplementary material for: Optimizing Microsatellite Marker Panels for Genetic Diversity and Population Genetic Studies: An Ant Colony Algorithm Approach with Polymorphic Information Content
Source: Biology (Basel). 2023 Sep 25;12(10):1280. doi: 10.3390/biology12101280 (PMC10604496; doi:10.3390/biology12101280)
Supplement: Supplementary file 1 [file biology-12-01280-s001.zip › Tables S1-S5.pdf]

Table S1. Summary of microsatellite markers used in this study.

Dataset	Locus	Size range	N_a[1]	N_{ea}[2]	*AR*[3]	*PIC*[4]	H_o[5]	H_e[6]
Gallus gallus 28 microsatellite markers	MCW0248	208–224	10	5.27	0.02	0.77	0.63	0.81
	MCW0111	80–120	31	9.77	0.05	0.88	0.65	0.90
	ADL0268	106–135	17	7.60	0.03	0.85	0.65	0.87
	LEI0234	212–376	72	26.22	0.12	0.95	0.76	0.96
	MCW0206	207–248	27	6.25	0.04	0.81	0.66	0.84
	MCW0034	201–249	27	9.81	0.04	0.88	0.62	0.90
	MCW0222	214–230	13	4.22	0.02	0.69	0.30	0.76
	MCW0103	259–270	9	3.68	0.01	0.64	0.50	0.73
	MCW0016	126–177	34	8.73	0.05	0.87	0.81	0.89
	LEI0166	245–365	21	5.17	0.03	0.74	0.57	0.81
	MCW0037	147–160	9	3.55	0.01	0.65	0.54	0.72
	MCW0295	82–111	18	7.32	0.03	0.84	0.67	0.86
	LEI0094	231–283	41	18.29	0.07	0.93	0.68	0.95
	MCW0098	215–267	10	1.68	0.02	0.31	0.28	0.41
	MCW0078	133–154	15	5.51	0.02	0.77	0.53	0.82
	MCW0081	102–157	26	4.40	0.04	0.73	0.61	0.77
	LEI0192	251–490	82	16.83	0.13	0.93	0.68	0.94
	MCW0014	160–217	25	6.55	0.04	0.79	0.40	0.85
	MCW0183	286–361	38	6.79	0.06	0.83	0.58	0.85
	ADL0278	109–151	25	7.70	0.04	0.84	0.64	0.87
	MCW0067	170–184	10	5.24	0.02	0.75	0.67	0.81
	ADL0112	112–144	17	5.58	0.03	0.77	0.57	0.82
	MCW0216	129–187	30	6.21	0.05	0.80	0.54	0.84
	MCW0104	186–254	46	10.08	0.08	0.88	0.66	0.90
	MCW0123	76–114	21	10.31	0.03	0.88	0.80	0.90
	MCW0330	247–291	25	8.36	0.04	0.85	0.60	0.88
	MCW0165	106–129	16	3.82	0.03	0.69	0.61	0.74
	MCW0069	152–186	20	8.31	0.03	0.86	0.70	0.88
Naemorhedus griseus 11 microsatellite markers	SY434F	76–97	8	1.64	0.10	0.37	0.08	0.39
	SY14F	86–125	11	2.51	0.14	0.57	0.22	0.60
	SY259F	211–218	5	1.14	0.06	0.12	0.00	0.12
	SY12BF	111–139	13	5.28	0.16	0.79	0.35	0.81
	SY93F	104–168	13	2.84	0.16	0.62	0.38	0.65
	SY129F	116–140	8	1.50	0.10	0.32	0.06	0.33
	SY76F	129–159	5	1.49	0.06	0.31	0.05	0.33
	SY449F	194–200	5	1.48	0.06	0.31	0.05	0.32
	SY128F	134–168	11	4.74	0.14	0.77	0.58	0.79
	SY84BF	162–202	10	1.65	0.13	0.38	0.19	0.39
	SY84F	161–185	5	2.06	0.06	0.47	0.06	0.51

[1] N_a: Number of alleles, [2] N_{ea}: Number of effective alleles, [3] *AR*: Allele richness, [4] *PIC*: Polymorphic information content, [5] H_o: Observed heterozygosity, [6] H_e: Expected heterozygosity.

Table S2. Summary of microsatellite markers selected by the *PIC*+ACO selection scheme according to various margin errors. Data include number of alleles (N_a), effective number of alleles (N_{ea}), allele richness (*AR*), polymorphic information content (*PIC*), and observed (H_o) and expected heterozygosity (H_e).

Dataset	AGD Accuracy loss tolerance	Locus	N_a	N_{ea}	*AR*	*PIC*	H_o	H_e
Gallus gallus 28 microsatellite markers	GGA_{10} (7 markers)	MCW0111	32	9.77	0.05	0.88	0.65	0.90
		LEI0234	73	26.22	0.12	0.95	0.76	0.96
		MCW0034	28	9.81	0.04	0.88	0.62	0.90
		MCW0016	35	8.73	0.05	0.87	0.81	0.89
		LEI0192	83	16.83	0.13	0.93	0.68	0.94
		MCW0183	39	6.79	0.06	0.83	0.58	0.85
		MCW0104	47	10.08	0.08	0.88	0.66	0.90
	GGA_5 (12 markers)	MCW0111	32	9.77	0.05	0.88	0.65	0.90
		LEI0234	73	26.22	0.12	0.95	0.76	0.96
		MCW0206	28	6.25	0.04	0.81	0.66	0.84
		MCW0034	28	9.81	0.04	0.88	0.62	0.90
		LEI0166	22	5.17	0.03	0.74	0.57	0.81
		LEI0094	42	18.29	0.07	0.93	0.68	0.95
		LEI0192	83	16.83	0.13	0.93	0.68	0.94
		MCW0183	39	6.79	0.06	0.83	0.58	0.85
		MCW0104	47	10.08	0.08	0.88	0.66	0.90
		MCW0123	22	10.31	0.03	0.88	0.80	0.90
		MCW0165	17	3.82	0.03	0.69	0.61	0.74
		MCW0069	21	8.31	0.03	0.86	0.70	0.88
	GGA_1 (26 markers)	MCW0248	11	5.27	0.02	0.77	0.63	0.81
		MCW0111	32	9.77	0.05	0.88	0.65	0.90
		ADL0268	18	7.60	0.03	0.85	0.65	0.87
		LEI0234	73	26.22	0.12	0.95	0.76	0.96
		MCW0206	28	6.25	0.04	0.81	0.66	0.84
		MCW0034	28	9.81	0.04	0.88	0.62	0.90
		MCW0222	14	4.22	0.02	0.69	0.30	0.76
		MCW0103	10	3.68	0.01	0.64	0.50	0.73
		MCW0016	35	8.73	0.05	0.87	0.81	0.89
		LEI0166	22	5.17	0.03	0.74	0.57	0.81
		MCW0037	10	3.55	0.01	0.65	0.54	0.72
		MCW0295	19	7.32	0.03	0.84	0.67	0.86
		MCW0098	11	1.68	0.02	0.31	0.28	0.41
		MCW0078	16	5.51	0.02	0.77	0.53	0.82
		MCW0081	27	4.40	0.04	0.73	0.61	0.77
		LEI0192	83	16.83	0.13	0.93	0.68	0.94
		MCW0014	26	6.55	0.04	0.79	0.40	0.85
		MCW0183	39	6.79	0.06	0.83	0.58	0.85
		ADL0278	26	7.70	0.04	0.00	0.64	0.87
		MCW0067	11	5.24	0.02	0.75	0.67	0.81
		ADL0112	18	5.58	0.03	0.77	0.57	0.82
		MCW0216	31	6.21	0.05	0.80	0.54	0.84
		MCW0104	47	10.08	0.08	0.88	0.66	0.90
		MCW0330	26	8.36	0.04	0.85	0.60	0.88
		MCW0165	17	3.82	0.03	0.69	0.61	0.74
		MCW0069	21	8.31	0.03	0.86	0.70	0.88

Naemorhedus griseus 11 microsatellite markers	NGR_{10} (6 markers)	SY434F	8	1.64	0.10	0.37	0.08	0.39
		SY14F	11	2.51	0.14	0.57	0.22	0.60
		SY12BF	13	5.28	0.16	0.79	0.35	0.81
		SY129F	8	1.50	0.10	0.32	0.06	0.33
		SY449F	5	1.48	0.06	0.31	0.05	0.32
		SY128F	11	4.74	0.14	0.77	0.58	0.79
	NGR_5 (9 markers)	SY434F	8	1.64	0.10	0.37	0.08	0.39
		SY14F	11	2.51	0.14	0.57	0.22	0.60
		SY12BF	13	5.28	0.16	0.79	0.35	0.81
		SY93F	13	2.84	0.16	0.62	0.38	0.65
		SY129F	8	1.50	0.10	0.32	0.06	0.33
		SY76F	5	1.49	0.06	0.31	0.05	0.33
		SY449F	5	1.48	0.06	0.31	0.05	0.32
		SY84BF	10	1.65	0.13	0.38	0.19	0.39
		SY84F	5	2.06	0.06	0.47	0.06	0.51
	NGR_1 (11 markers)	SY434F	8	1.64	0.10	0.37	0.08	0.39
		SY14F	11	2.51	0.14	0.57	0.22	0.60
		SY259F	5	1.14	0.06	0.12	0.00	0.12
		SY12BF	13	5.28	0.16	0.79	0.35	0.81
		SY93F	13	2.84	0.16	0.62	0.38	0.65
		SY129F	8	1.50	0.10	0.32	0.06	0.33
		SY76F	5	1.49	0.06	0.31	0.05	0.33
		SY449F	5	1.48	0.06	0.31	0.05	0.32
		SY128F	11	4.74	0.14	0.77	0.58	0.79
		SY84BF	10	1.65	0.13	0.38	0.19	0.39
		SY84F	5	2.06	0.06	0.47	0.06	0.51

Table S3. Statistical comparison between the most accurate selection method and the random microsatellite selection scheme.

Gallus gallus 28 microsatellite markers dataset				*Naemorhedus griseus* 11 microsatellite markers dataset			
Quantity of microsatellite	Best selection method	*p*-value	Mean difference	Quantity of microsatellite	Best selection method	*p*-value	Mean difference
27	*PIC* + ACO[1]	< 0.001	0.002	10	ACO[2]	< 0.001	0.039
26	*PIC* + ACO[1]	< 0.001	0.003	9	*PIC* + ACO[1]	< 0.001	0.029
25	*PIC* + ACO[1]	< 0.001	0.002	8	ACO[2]	< 0.001	0.012
24	*PIC* + ACO[1]	< 0.001	0.002	7	*PIC* + ACO[1]	< 0.001	0.008
23	*PIC* + ACO[1]	< 0.001	0.003	6	Random[4]	–	0.000
22	*PIC* + ACO[1]	< 0.001	0.003	5	Random[4]	–	0.000
21	*PIC* + ACO[1]	< 0.001	0.004	4	*PIC* + ACO[1]	ns[5]	0.005
20	*PIC* + ACO[1]	< 0.001	0.004	3	Random[4]	–	0.000
19	*PIC* + ACO[1]	< 0.001	0.005	2	Random[4]	–	0.000
18	*PIC* + ACO[1]	< 0.001	0.006				
17	*PIC* + ACO[1]	< 0.001	0.005				
16	*PIC* + ACO[1]	< 0.001	0.007				
15	*PIC* + ACO[1]	< 0.001	0.007				
14	*PIC* + ACO[1]	< 0.001	0.006				
13	*PIC* + ACO[1]	< 0.001	0.009				
12	*PIC* + ACO[1]	< 0.001	0.010				
11	*PIC* + ACO[1]	< 0.001	0.013				
10	*PIC* + ACO[1]	< 0.001	0.013				
9	*PIC* + ACO[1]	< 0.001	0.014				
8	*PIC* + ACO[1]	< 0.001	0.020				
7	*PIC* + ACO[1]	< 0.001	0.035				
6	*PIC* + ACO[1]	< 0.001	0.039				
5	ACO[2]	< 0.001	0.067				
4	*PIC*[3]	< 0.001	0.062				
3	*PIC* + ACO[1]	< 0.001	0.127				
2	*PIC* + ACO[1]	< 0.001	0.273				

[1] *PIC* + ACO, selection scheme involving ranking the markers by their polymorphic information content and subsequently optimizing the set using *PIC* + ACO algorithm.
[2] ACO, selection scheme using only the ant colony optimization algorithm without any prior information on the *PIC* of the markers.
[3] *PIC*, selection scheme sorting microsatellites on their *PIC* and selecting the most informative loci.
[4] Random, selection scheme selecting randomly microsatellite markers.
[5] ns, not statistically significant ($p > 0.05$).

Table S4. Number of population cluster estimated by the Structure software (Evanno et al., 2005).

Gallus gallus 28 microsatellite markers dataset		*Naemorhedus griseus* 11 microsatellite markers dataset	
Number of loci	Quantity of cluster	Number of loci	Quantity of cluster
28	2	11	2
27	2	10	2
26	2	9	2
25	2	8	4
24	2	7	2
23	2	6	2
22	2	5	3
21	2	4	3
20	2	3	7
19	2	2	2
18	2		
17	2		
16	2		
15	2		
14	2		
13	2		
12	2		
11	2		
10	2		
9	2		
8	2		
7	2		
6	2		
5	2		
4	2		
3	2		
2	2		

References

Evanno, G., Regnaut, S., & Goudet, J. (2005). Detecting the number of clusters of individuals using the software structure: a simulation study. *Molecular Ecology*, 14, 2611–2620. https://doi.org/10.1111/j.1365-294x.2005.02553.x

Table S5. Clustering of each subpopulations by using the Bayesian clustering of the Structure software (Evanno et al., 2005).

K	N	Population clusters[1] (*p*-value[2]) A	B
2	28	SiSaKet (< 0.001), HuaiSai_Ggs (< 0.001), KP (< 0.001), DECOY (< 0.001), RoiEt (< 0.001), ChaingRai (< 0.001), DT (< 0.001), KhokMaiRua (< 0.001), HuaiYangPan (< 0.001), KhaoKho (< 0.001), Petchburi (< 0.001), PHD (< 0.001), Songkhla2 (< 0.001), HuaiSai_Gg (< 0.001), CH (< 0.001), Chaiyaphum (< 0.001), BLWF (< 0.001), FightChick (< 0.001), Songkhla1 (< 0.001), Sa_Kaeo (< 0.001), ChiangMaiZoo (< 0.001), BLBF (< 0.001), BT (< 0.001), Chabthaburi (< 0.001), LHK (< 0.001)	Lamphun (< 0.001), Chiang Rai (< 0.001), khonkan (< 0.001), MHS (< 0.001), Udonthani (< 0.001), Mae Hong Son (< 0.001), Nakhon Prathom (< 0.001)
	26 (GGA_1)	SiSaKet (< 0.001), HuaiSai_Ggs (< 0.001), KP (< 0.001), DECOY (< 0.001), RoiEt (< 0.001), ChaingRai (< 0.001), DT (< 0.001), KhokMaiRua (< 0.001), HuaiYangPan (< 0.001), KhaoKho (< 0.001), khonkan (< 0.001), Petchburi (< 0.001), PHD (< 0.001), Songkhla2 (< 0.001), HuaiSai_Gg (< 0.001), CH (< 0.001), Chaiyaphum (< 0.001), BLWF (< 0.001), FightChick (< 0.001), Songkhla1 (< 0.001), Sa_Kaeo (< 0.001), ChiangMaiZoo (< 0.001), BLBF (< 0.001), BT (< 0.001), Chabthaburi (< 0.001), LHK (< 0.001)	Lamphun (< 0.001), Chiang Rai (< 0.001), MHS (< 0.001), Udonthani (< 0.001), Mae Hong Son (< 0.001), Nakhon Prathom (< 0.001)
	12 (GGA_5)	SiSaKet (< 0.001), HuaiSai_Ggs (< 0.001), KP (< 0.001), DECOY (< 0.001), RoiEt (< 0.001), ChaingRai (< 0.001), DT (< 0.001), KhokMaiRua (< 0.001), HuaiYangPan (< 0.001), KhaoKho (< 0.001), Petchburi (< 0.001), PHD (< 0.001), Songkhla2 (< 0.001), HuaiSai_Gg (< 0.001), CH (< 0.001), Chaiyaphum (< 0.001), BLWF (< 0.001), FightChick (< 0.001), Songkhla1 (< 0.001), Sa_Kaeo (< 0.001), ChiangMaiZoo (< 0.001), BLBF (< 0.001), BT (< 0.001), Chabthaburi (< 0.001), LHK (< 0.001)	Lamphun (< 0.001), Chiang Rai (< 0.001), khonkan (< 0.001), MHS (< 0.001), Udonthani (< 0.001), Mae Hong Son (< 0.001), Nakhon Prathom (< 0.001)
	7 (GGA_{10})	SiSaKet (< 0.001), HuaiSai_Ggs (< 0.001), KP (< 0.001), DECOY (< 0.001), RoiEt (< 0.001), ChaingRai (< 0.001), DT (< 0.001), KhokMaiRua (< 0.001), HuaiYangPan (< 0.001), KhaoKho (< 0.001), Petchburi (< 0.001), PHD (< 0.001), Songkhla2 (< 0.001), HuaiSai_Gg (< 0.001), CH (< 0.001), Chaiyaphum (< 0.001), BLWF (< 0.001), FightChick (< 0.001), Songkhla1 (< 0.001), Sa_Kaeo (< 0.001), ChiangMaiZoo (< 0.001), BLBF (< 0.001), BT (< 0.001), Chabthaburi (< 0.001), LHK (< 0.001)	Lamphun (< 0.001), Chiang Rai (< 0.001), khonkan (< 0.001), MHS (< 0.001), Udonthani (< 0.001), Mae Hong Son (< 0.001), Nakhon Prathom (< 0.001)

		I	II	III	IV	V	VI	VII
7	28	HuaiSai_Ggs (< 0.001), KP (< 0.001), DECOY (< 0.001), KhokMaiRua (< 0.001), KhaoKho (< 0.001), Petchburi (< 0.001), PHD (< 0.001), Songkhla2 (ns), HuaiSai_Gg (< 0.001), CH (< 0.001), FightChick (< 0.001), Songkhla1 (ns), LHK (< 0.001)	Lamphun (< 0.001), Chiang Rai (< 0.001), Udonthani (< 0.001), Mae Hong Son (< 0.01), Nakhon Prathom (< 0.001)	DT (ns), BLWF (< 0.001), BLBF (< 0.001), BT (< 0.001)	ChaingRai (< 0.001), khonkan (< 0.001), Chaiyaphum (< 0.001), ChiangMaiZoo (< 0.001)	SiSaKet (< 0.001), RoiEt (< 0.001), HuaiYangPan (< 0.001)	Sa_Kaeo (< 0.001), Chabthaburi (< 0.001)	MHS (< 0.001)
	26 (GGA_1)	HuaiSai_Ggs (< 0.001), KP (< 0.001), DECOY (< 0.001), KhokMaiRua (< 0.001), KhaoKho (< 0.001), Petchburi (< 0.001), PHD (< 0.001), Songkhla2 (ns), HuaiSai_Gg (< 0.001), CH (< 0.001), FightChick (< 0.001), Songkhla1 (< 0.001), ChiangMaiZoo (ns), LHK (< 0.001)	Lamphun (< 0.001), Chiang Rai (< 0.001), Udonthani (< 0.001), Mae Hong Son (< 0.001), Nakhon Prathom (< 0.001)	DT (< 0.5), BLWF (< 0.001), BLBF (< 0.001), BT (< 0.001)	ChaingRai (ns), khonkan (< 0.001)	SiSaKet (< 0.001), RoiEt (< 0.001), HuaiYangPan (< 0.001)	Chaiyaphum (< 0.001), Sa_Kaeo (< 0.001), Chabthaburi (< 0.001)	MHS (< 0.001)
	12 (GGA_5)	HuaiSai_Ggs (< 0.001), KP (< 0.001), DECOY (< 0.001), DT (< 0.001), KhokMaiRua (< 0.001), HuaiYangPan (< 0.001), Petchburi (< 0.01), PHD (< 0.001), Songkhla2 (ns), HuaiSai_Gg (< 0.001), CH (< 0.001), FightChick (< 0.001), Songkhla1 (< 0.001), ChiangMaiZoo (ns), LHK (< 0.001)	Lamphun (< 0.001), MHS (< 0.001), Udonthani (< 0.001), Mae Hong Son (< 0.001), Nakhon Prathom (< 0.001)	BLWF (< 0.001), BLBF (< 0.001), BT (< 0.001)	Chiang Rai (< 0.001), khonkan (< 0.001)	SiSaKet (< 0.001), RoiEt (< 0.001), KhaoKho (< 0.001)	Sa_Kaeo (< 0.001), Chabthaburi (< 0.001)	ChaingRai (< 0.001), Chaiyaphum (< 0.001)
	7 (GGA_{10})	KP (< 0.001), DECOY (< 0.001), HuaiYangPan (< 0.001), Songkhla2 (ns), CH (< 0.01), FightChick (< 0.001), Songkhla1 (ns), ChiangMaiZoo (ns), LHK (< 0.001)	Lamphun (< 0.001), Chiang Rai (< 0.001), MHS (< 0.001), Udonthani (< 0.001), Mae Hong Son (< 0.001), Nakhon Prathom (< 0.001)	HuaiSai_Gg (ns), BT (< 0.001)	HuaiSai_Ggs (< 0.001), DT (< 0.001), KhokMaiRua (< 0.5), Petchburi (< 0.001), PHD (ns), BLWF (< 0.001), BLBF (< 0.001)	SiSaKet (< 0.001), RoiEt (< 0.001), KhaoKho (< 0.001)	Sa_Kaeo (< 0.001), Chabthaburi (< 0.001)	ChaingRai (< 0.5), khonkan (< 0.001), Chaiyaphum (< 0.001)

		a	b	c	d	e	f	g	h	i
9	28	KP (< 0.001), DECOY (< 0.001), ChaingRai (< 0.001), PHD (< 0.001), Songkhla2 (< 0.001), HuaiSai_Gg (ns), CH (< 0.001), FightChick (< 0.001), Songkhla1 (< 0.001), ChiangMaiZoo (< 0.001), LHK (< 0.001)	Lamphun (< 0.001), Chiang Rai (< 0.001), MHS (< 0.001), Udonthani (< 0.001), Mae Hong Son (< 0.001), Nakhon Prathom (< 0.001)	HuaiSai_Ggs (< 0.5), KhokMaiRua (< 0.001), KhaoKho (< 0.01), Petchburi (< 0.001)	DT (< 0.01), BLWF (< 0.001), BLBF (< 0.001), BT (< 0.001)	Sa_Kaeo (< 0.001), Chabthaburi (< 0.001)	SiSaKet (< 0.001), RoiEt (< 0.001)	HuaiYangPan (< 0.001)	Chaiyaphum (< 0.001)	khonkan (< 0.001)
	26 (GGA_1)	KP (< 0.001), DECOY (< 0.001), PHD (< 0.001), CH (< 0.001), FightChick (< 0.001), LHK (< 0.001)	ChaingRai (< 0.001), khonkan (< 0.001), Songkhla2 (ns), Chaiyaphum (< 0.001), Songkhla1 (ns), ChiangMaiZoo (< 0.01)	HuaiSai_Ggs (< 0.001), KhokMaiRua (< 0.5), HuaiYangPan (< 0.001), Petchburi (< 0.001), HuaiSai_Gg (< 0.01)	DT (< 0.01), BLWF (< 0.001), BLBF (< 0.001), BT (< 0.001)	Sa_Kaeo (< 0.001), Chabthaburi (< 0.001)	SiSaKet (< 0.001), RoiEt (< 0.001)	Lamphun (< 0.001), MHS (< 0.001), Udonthani (< 0.001), Mae Hong Son (< 0.001), Nakhon Prathom (< 0.001)	Chiang Rai (< 0.001)	KhaoKho (< 0.001)
	12 (GGA_5)	Lamphun (< 0.001), Udonthani (< 0.001), Mae Hong Son (< 0.001), Nakhon Prathom (< 0.001)	MHS (< 0.001)	SiSaKet (< 0.001), RoiEt (< 0.001), Chaiyaphum (< 0.001)	Chiang Rai (< 0.001), khonkan (< 0.001)	BLWF (< 0.001), BLBF (< 0.001), BT (< 0.001)	ChaingRai (< 0.001), Songkhla1 (< 0.5), ChiangMaiZoo (< 0.001), Chabthaburi (< 0.001)	KhokMaiRua (< 0.001), KhaoKho (< 0.001), Songkhla2 (ns)	HuaiSai_Ggs (< 0.001), DT (< 0.01), HuaiYangPan (< 0.001), Petchburi (ns), HuaiSai_Gg (< 0.5)	KP (< 0.001), DECOY (< 0.01), PHD (< 0.001), CH (< 0.001), FightChick (< 0.001), Sa_Kaeo (< 0.001), LHK (< 0.001)
	7 (GGA_{10})	KP (< 0.001), DECOY (< 0.001), KhokMaiRua (< 0.001), Petchburi (< 0.01), PHD (< 0.001), Songkhla2 (ns), CH (< 0.001), FightChick (< 0.001), Songkhla1 (< 0.001), ChiangMaiZoo (< 0.5), LHK (< 0.001)	HuaiSai_Ggs (< 0.001), DT (< 0.001), HuaiSai_Gg (< 0.01), BLWF (< 0.001), BLBF (< 0.001), BT (< 0.001)	Lamphun (< 0.001), MHS (< 0.001), Udonthani (< 0.001), Mae Hong Son (< 0.001), Nakhon Prathom (< 0.001)	SiSaKet (< 0.001), RoiEt (< 0.001)	Chabthaburi (< 0.001)	Chiang Rai (< 0.001), khonkan (< 0.001)	HuaiYangPan (< 0.001)	ChaingRai (ns), KhaoKho (< 0.001), Chaiyaphum (< 0.001)	Sa_Kaeo (< 0.001)

[1] According to the value of *K* chosen for the Bayesian clustering algorithm, population clusters are named A, and B for *K*=2 ; I, II, III, IV, V, VI, and VII for *K*=7 ; and a, b, c, d, e, f, g, h, and i for *K*=9.

[2] The *p*-value was measured by independent *t*-test on the posterior probability.

References

Evanno, G., Regnaut, S., & Goudet, J. (2005). Detecting the number of clusters of individuals using the software structure: a simulation study. *Molecular Ecology*, 14, 2611–2620. https://doi.org/10.1111/j.1365-294x.2005.02553.x
