# Supplementary figures and images for: Optimizing Microsatellite Marker Panels for Genetic Diversity and Population Genetic Studies: An Ant Colony Algorithm Approach with Polymorphic Information Content
Source: Biology (Basel). 2023 Sep 25;12(10):1280. doi: 10.3390/biology12101280 (PMC10604496; doi:10.3390/biology12101280)

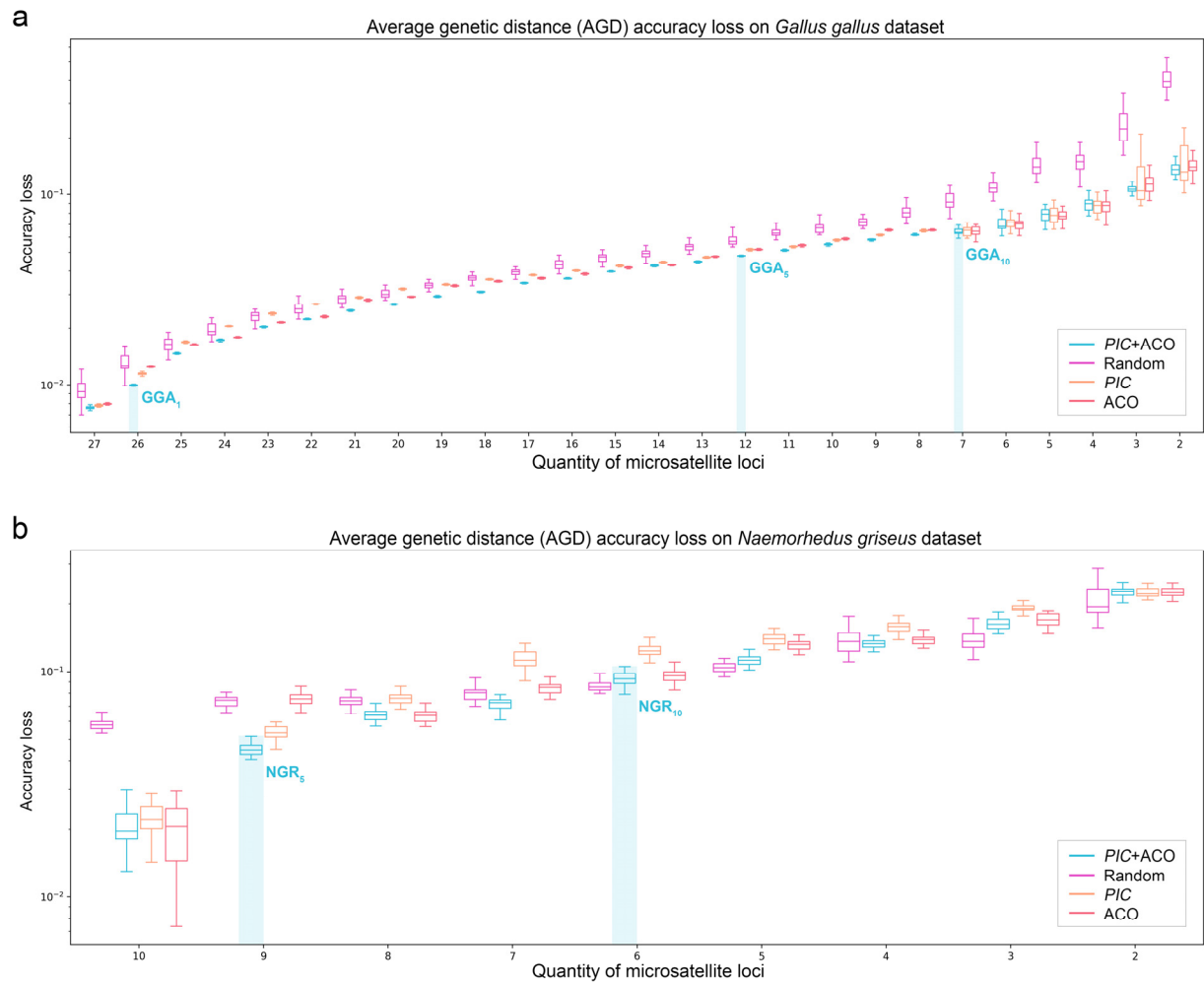

**Figure S1.** (Rasoarahona et al.)



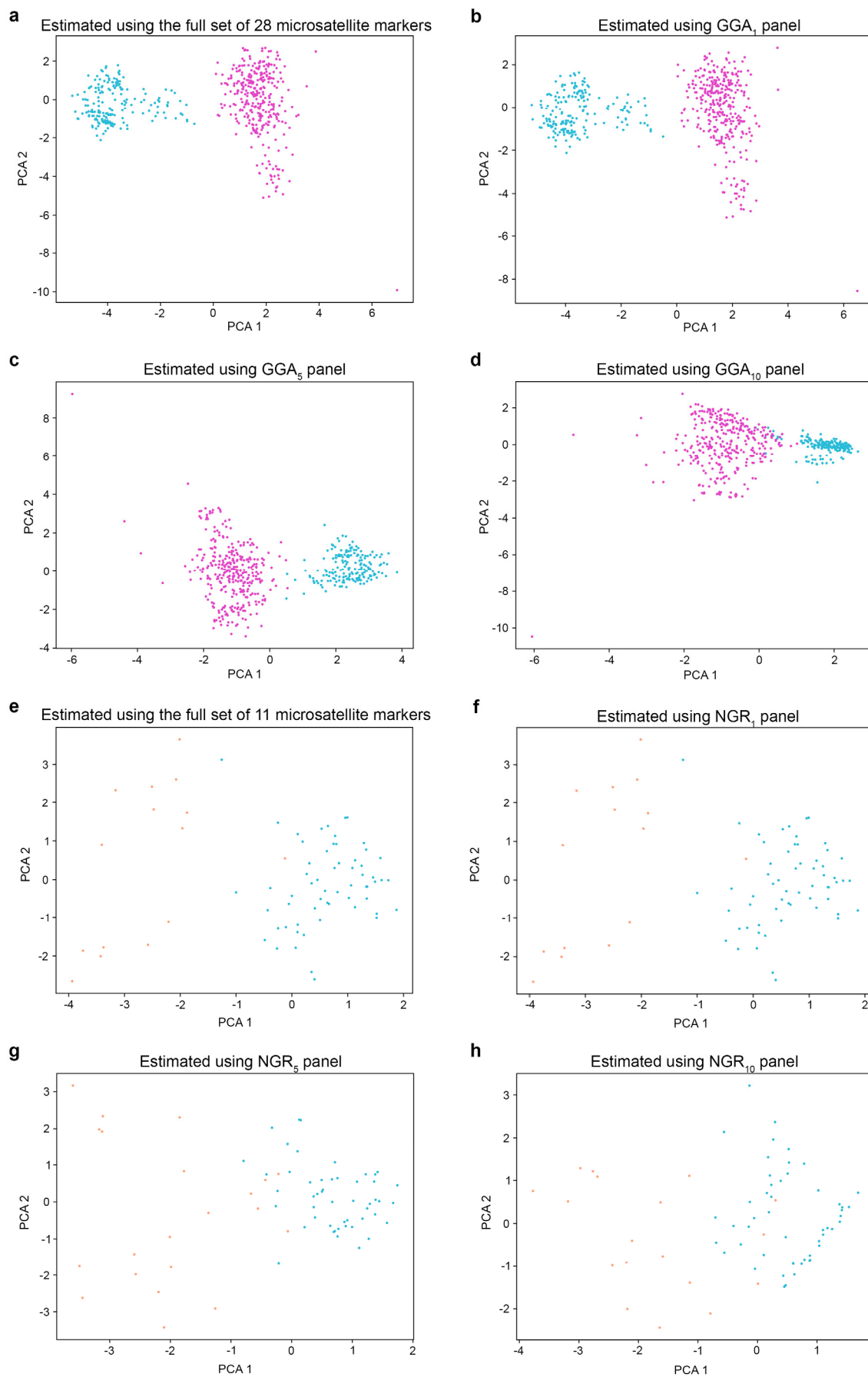

**Figure S3.** (Rasoarahona et al.)

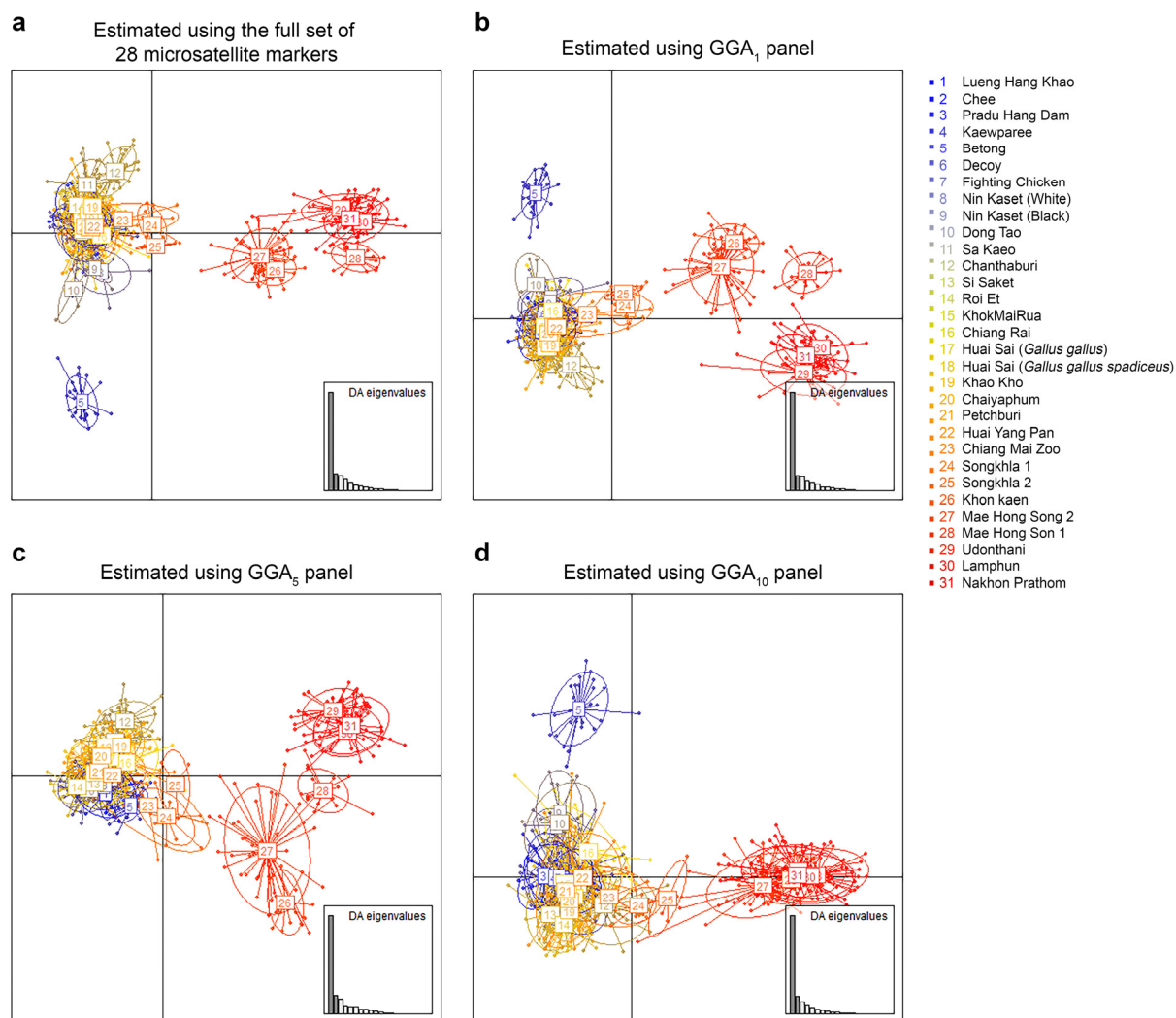

**Figure S4.** (Rasoarahona et al.)

Supplement: Supplementary file 1 [file biology-12-01280-s001.zip › Figure S1-S4.pdf]
